# Letter to the editor

**DOI:** 10.1186/s13049-024-01199-w

**Published:** 2024-04-15

**Authors:** Dag Ferner Netteland, Pål Aksel Næss, Christine Gaarder, Eirik Helseth

**Affiliations:** 1 Department of Neurosurgery, Oslo University Hospital, Pb 4956, 0424 Nydalen, Oslo, Norway; 2 Faculty of Medicine, University of Oslo, Oslo, Norway; 3 Department of Traumatology, Oslo University Hospital, Oslo, Norway

Dear Editor,

We read the meta-analysis on tranexamic acid (TXA) in traumatic brain injury (TBI) by Song et al. with great interest [1]. Whether TBI patients benefit from TXA has been a matter of controversy since CRASH-3, the largest study to date addressing this question [2]. In CRASH-3, the primary outcome was defined as head-injury-related mortality at 28 days post trauma and was revised during the course of the study.

We note that the pooled analysis for mortality by Song et al. is based on the exact same included studies as in the previously published meta-analysis by Lawati et al. from 2021, yet we also note that the results differ, and conclusions are diverging [3]. In Lawati et al., all-cause mortality was chosen for the pooled analysis based on a concern for misclassification issues; also CRASH-3 was the only study out of the included that reported head-injury-related mortality.

The concern for misclassification issues is also touched upon in the discussion by Song et al., and it is stated that “the mortality mentioned in the article (CRASH 3) refers to all-cause mortality”. It is however unclear to us what definition of mortality was used in their pooled mortality analysis, as the data included from CRASH-3 seem to correlate with the primary outcome analysis using head-injury-related mortality in the included forest plot (Fig. 3, Song et al.). Moreover, we note that the included number of subjects from the same eight studies differ between the pooled analyses for mortality of the two meta-analyses. Lastly, we note a discrepancy between the conclusion of TXA leading to a reduced mortality rate in TBI based on RR = 0.92, 95%CI 0.85–1.00; *p* = 0.05, and a stated significance level of *p* < 0.05 by Song et al.

We hope this letter will lead to clarification of the questions raised above and encourage the authors to contextualize their results with the results of the meta-analysis by Lawati et al.
